# A common timing mechanism across different millisecond domains: evidence from perceptual and motor tasks

**DOI:** 10.1038/s41598-023-48238-7

**Published:** 2023-11-29

**Authors:** Magdalena Stanczyk, Elzbieta Szelag, Klaudia Krystecka, Aneta Szymaszek

**Affiliations:** Nencki Institute of Experimental Biology, Polish Academy of Sciences, Warsaw, Poland

**Keywords:** Human behaviour, Perception

## Abstract

Temporal information processing (TIP) constitutes a complex construct that underlies many cognitive functions and operates in a few hierarchically ordered time domains. This study aimed to verify the relationship between the tens of milliseconds and hundreds of milliseconds domains, referring to perceptual and motor timing, respectively. Sixty four young healthy individuals participated in this study. They underwent two auditory temporal order judgement tasks to assess their performance in the tens of milliseconds domain; on this basis, groups of high-level performers (HLP) and low-level performers (LLP) were identified. Then, a maximum tapping task was used to evaluate performance in the hundreds of milliseconds domain. The most remarkable result was that HLP achieved a faster tapping rate and synchronised quicker with their “internal clock” during the tapping task than did LLP. This result shows that there is a relationship between accuracy in judging temporally asynchronous stimuli and ability to achieve and maintain the pace of a movement adequate to one’s internal pacemaker. This could indicate the strong contribution of a common timing mechanism, responsible for temporal organisation and coordination of behaviours across different millisecond domains.

## Introduction

Time is believed to be a fundamental basis for our cognition. Extensive research studies indicate that temporal information processing (TIP) underpins many cognitive functions, such as memory, learning, attention, motor activities, planning, perception, and decision making, thereby playing a central role in our everyday activities^1–5^. Adjusting behaviours to a constantly changing environment requires the existence of a hypothetical internal clock responsible for processing temporal information extracted from different sensory modalities across various time domains^1,2,6^. The regulation of the speed of this hypothesised internal clock, whose effectiveness is based on the speed of the internal pacemaker, varies between individuals, leading to a faster clock for some and a slower clock for others. Thus, individuals differ in the speed of their internal pacemaker, which influences their tempo and, consequently, the effectiveness with which they process information.

According to the model proposed by Pöppel^7^, TIP is not a monolithic entity and a few hierarchically ordered time domains or operational processing windows can be distinguished. Pöppel’s model suggested the basic domain to be some tens of milliseconds. Further research extended this model and distinguished two domains on the millisecond level: the first of tens of milliseconds and the second of hundreds of milliseconds. These two domains pertain to different mental processes and can be studied with different experimental paradigms^8–10^. The present study focused on the relationship between the tens and the hundreds of milliseconds domains.

The first domain, operating over tens of milliseconds, is related to sequential analytical operations in which consecutive elements are identified and processed successfully. There are several experimental paradigms that assess individuals’ TIP ability in this domain, and the temporal-order judgement (TOJ) task is the most popular^11–14^. This measures the participant’s ability to perceive the order of paired stimuli separated by a silent gap of some tens of milliseconds. The participant’s ability is indexed by their temporal-order threshold value (TOT)—the minimum gap necessary to identify the before–after temporal relation of the presented stimuli^15,16^. According to the literature^17^, shorter TOT reflects better TIP performance in the tens of milliseconds domain. The TOJ task is categorised as *perceptual timing*, in which no movement is required and the stimulus is processed based only on its temporal features^11,18^. TOJ paradigms may apply various stimulus modalities (visual, auditory, tactile); however, auditory stimuli seem to be the most commonly used. Within the auditory TOJ task, various measurement procedures may be employed^11–13,17,19–22^. For example, in the spatial TOJ task, participants are presented monaurally with two identical stimuli (clicks or bursts) and the task is to identify the order of ears to which the first and second stimuli were delivered (i.e., left–right or right–left). In contrast, in the spectral TOJ task, two different stimuli are presented (e.g., high and low tones delivered binaurally) and the task is to indicate their order (high–low or low–high). Both TOJ paradigms are intended to measure unadulterated TIP ability in the tens of milliseconds domain, but they also involve nontemporal task-specific perceptual processes. Our study therefore used both spatial and spectral TOJ tasks. The overlapping of TIP performance in these two tasks increases the reliability of measurement of TIP ability on this processing level.

We also examined the hundreds of milliseconds domain, which is strongly related to motor performance^1^. The literature describes several paradigms for assessing TIP ability on this level, including temporal reproduction or discrimination^23^; however, the tapping task is the most widely used^24–26^. This task pertains to motor timing as it corresponds to the production of timed motor actions and coordination of movements^5^. There exist several types of tapping tasks. Participants could be instructed to tap with their finger in synchronisation with sequences of external events, such as isochronous sounds (synchronisation tapping task^25^). In a different task type, after the introductory phase in which participants synchronise their tapping with external sounds, participants are asked to continue tapping a series without any external cues (continuation tapping task); in another type, the participant taps according to their comfortable and preferred pace, without any time pressure (spontaneous tapping task). Previous researchers have shown that spontaneous motor tempo can be observed across a broad range of activities, including walking^27^. Most of the tapping tasks listed above highly engage the participant’s working memory^1^.

In our study, the maximum tapping task was implemented to reliably measure participants’ unadulterated motor timing performance, independent of working memory. In this task, participants are instructed to tap repeatedly as fast as they can. Most literature reports consider the inter-response interval (IRI)—the time interval from the onset of the one tap to the onset of the following tap—to be a crucial index in the tapping task^28,29^. A faster tapping rhythm reflects better temporal performance in the hundreds of milliseconds domain. There exists evidence that slowing of tapping rhythm is related to age, sex, cognitive impairments, brain injuries, and developmental disorders^24^. As far as we are aware, only a few studies^24,26,28^ refer to the Inter Tap Interval (ITI; the time interval between offset of one tap and onset of the following one) and key touching time (KTT; the time interval from the onset to the offset of a single tap). In addition to the IRI (the sum of the ITI and KTT), this study also focused on ITI and KTT, which are more precise indices. The ITI is considered to be a central timing component, responsible for mental planning and preparing for subsequent finger movements. For that reason, this component is believed to be based on a pacemaker, which may reveal the frequency of the hypothetical internal clock. On the other hand, KTT—the peripheral component—refers to the execution of consecutive finger movements. It is responsible for the integration of sensorimotor information^28,30,31^.

A considerable amount of literature has concentrated exclusively on TIP performance over particular time domains; however, the relationship between these domains, in particular with reference to the different timing systems, remains unclear. In our previous paper^2^, we provided evidence of associations between the tens of milliseconds domain (TOJ task) and the supra-second time domain (subjective accentuation task) using the auditory modality. The results suggested the existence of a common timing mechanism that is active independently of the tested time domains. In the present exploratory study, the relationships between two other time domains were investigated: the tens of milliseconds domain, with reference to the perception of the order of sounds, and the hundreds of milliseconds domain, with reference to the production of motor acts.

Overlapping of these domains may be expected from the theoretical point of view as well as from fMRI and electrophysiological studies. The hierarchical model of TIP assumes that performance on a low level (here, the tens of milliseconds domain) is involved in successful functioning at higher levels (here, the hundreds of milliseconds domain). Furthermore, neuroimaging studies have shown that TIP involves the interaction of multiple brain structures, including core timing areas—universal to both temporal domains and task modalities—as well as domain specific regions. The essential network of basic structures combines subcortical areas, such as the basal ganglia and thalamus, as well as cortical areas such as the supplementary motor area^32^^,^^33^. However, to understand how our brain processes time, it is not enough to identify the network of structures involved in this process—it is also necessary to characterise the complex dynamic interactions between these structures. Time intervals in the millisecond domain have been more often explored with magnetoencephalography (MEG) or electroencephalography (EEG) due to their high temporal resolution. Beta and gamma rhythms are considered to be electrophysiological candidates for a common timing mechanism. It has been found that spontaneous gamma band oscillations correspond with threshold values on the TOJ task (^34^; see “Discussion” Section). Furthermore, Bernasconi^35^ showed that beta oscillations are associated with perception of succession. Moreover, Bartolo and Merchant^36^ observed beta oscillations in tasks operating also in the hundreds of milliseconds domain. These studies may suggest the possible neural indicators for the existence of a common timing mechanism.

Thus, TIP acts across different time domains (tens vs hundreds of milliseconds domains) that may have similar neural backgrounds—the different timing systems (auditory perception vs motor performance) used here to engage these two time domains have been shown to be entangled^18,37^. Several studies, including neuroimaging and clinical ones, have observed interrelations between perceptual and motor timing. Some studies have indicated that motor timing is crucial for higher cognitive functions^5^; however, the efficiency of motor performance depends on accurate temporal judgements^38^. Kock et al.^39^ confirmed that the brain structures involved in motor actions are implicated in both motor and perceptual timing. Monier^18^ reported that the motor network is recruited to process timing even in the absence of movement. Furthermore, it has been shown that patients suffering from movement disorders (e.g., Tourette’s syndrome, essential brain tremor, cerebellar degeneration) exhibit timing impairments, even if the task does not require motor acts^39^. Referring to electrophysiological data, Wiener^40^ reported that beta oscillations exhibit a supra-modal property—that is to say, beta oscillations have commonly been associated with motor tasks as well as with perceptual timing.

Given the unclear relationships between TIP on the tens versus hundreds of milliseconds levels, the present study aims to explore whether a hypothetical common timing mechanism (i.e., internal clock) controls our performance on both millisecond domains, independently of timing system. Based on the hierarchical model of TIP as well as neuroimaging evidence of overlap between core timing structures, we expect that better TIP performance in the tens of milliseconds range, which is considered a basic domain, will be accompanied by better performance in the hundreds of milliseconds domain. Moreover, since our paradigms incorporate different task specificity (auditory vs motor), we would like to verify whether such cross-domain relationships are independent of timing system.

As far as we are aware, this is the first study considering cross-domain and cross-timing-system relations; we therefore adopted a more exploratory approach.

## Methods

### Participants

Sixty four adults (30 females and 34 males, aged between 20 and 27 years; M_age_ = 23 ± 2 years) participated in this study. They reported having no prior neurological or psychiatric disorders, as well as not taking substances that could affect the central nervous system. Moreover, all participants reported not having any formal musical education. Participants were screened for normal levels of cognitive abilities with the Raven Standard Progressive Matrices and for normal hearing using pure-tone audiometry (Audiometer MA33, MAICO). The study was in line with the Declaration of Helsinki and was approved by the Bioethics Committee of Nicolaus Copernicus University (permission no. KB 289/2019). All participants provided their written informed consent prior to the study.

### Procedure

**Figure 1:**
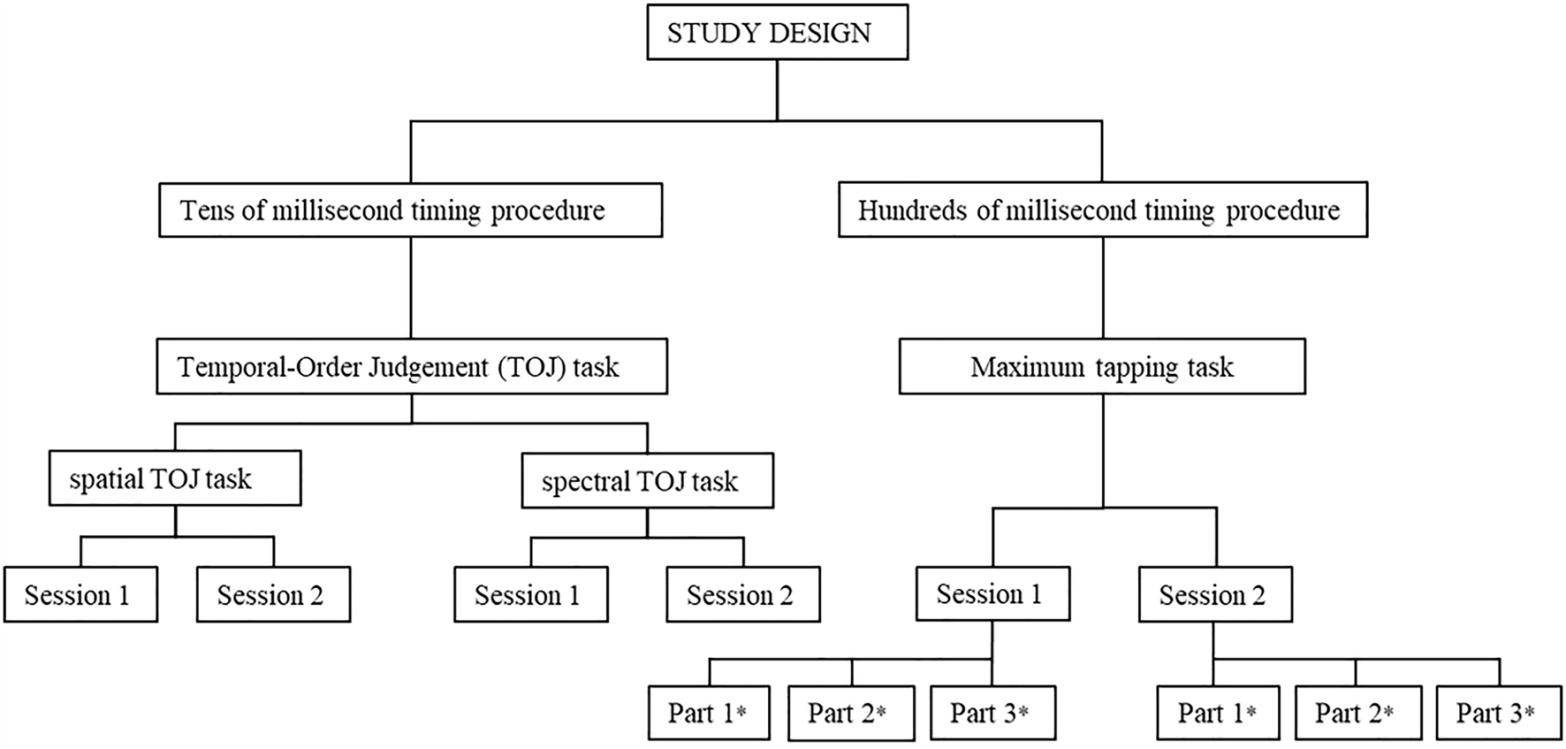
Schema of the study design. *The data obtained in each session were divided into three parts (Part 1, 2, and 3) at the stage of the analysis.

The experimental studies were conducted in a soundproof room at the Nencki Institute of Experimental Biology. To measure timing ability, two procedures were applied: the tens of milliseconds timing procedure used the TOJ task developed and tested in our previous reports^2,17^; the maximum tapping task was used for the hundreds of milliseconds timing procedure (see below for the schema of the study design, Fig. 1). The order of both timing procedures was the same for all subjects.

#### Tens of milliseconds timing procedure

**Figure 2:**
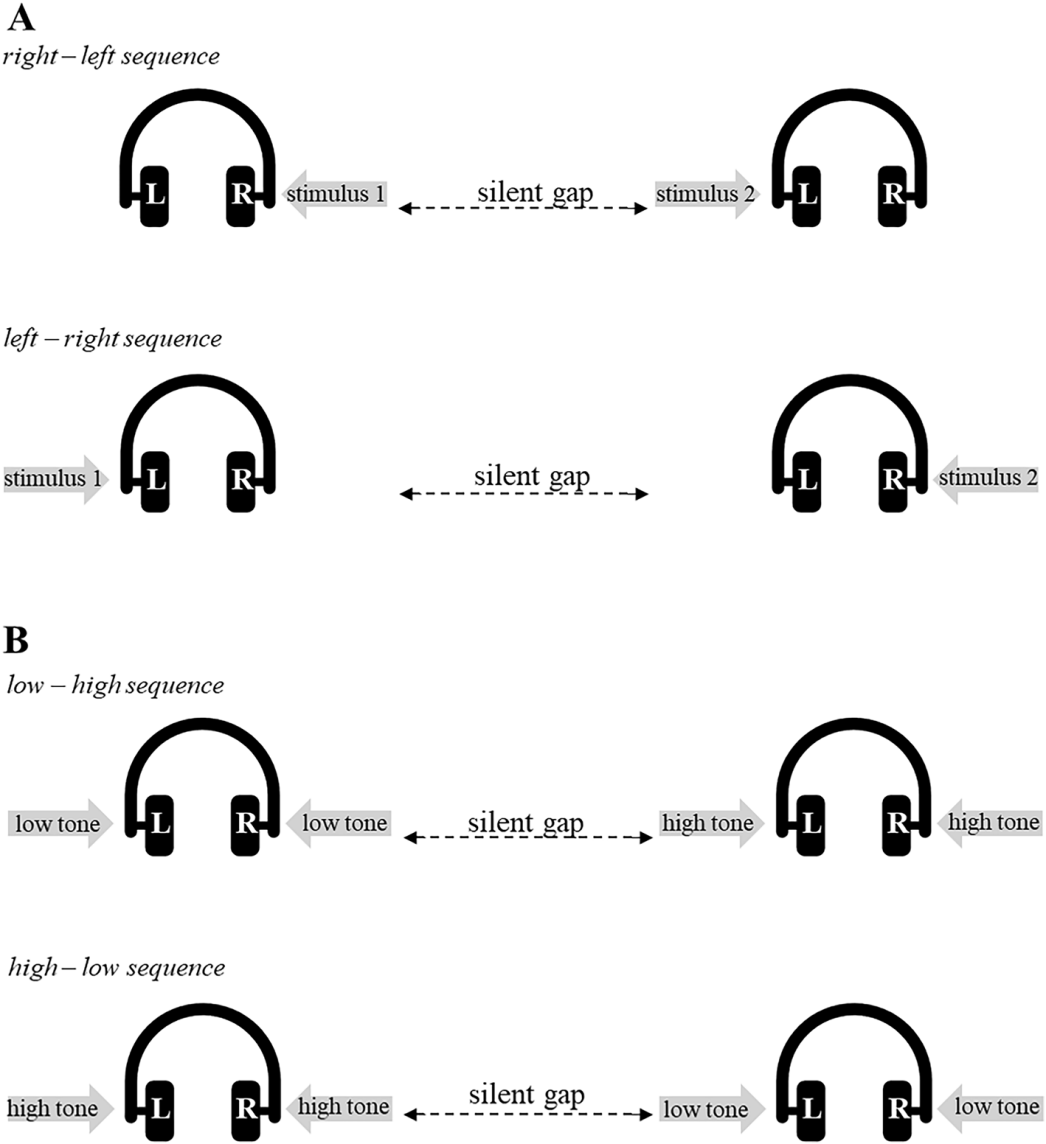
The experimental situation in the spatial (**A**) and spectral (**B**) TOJ tasks.

As this task was described in our previous reports, mentioned above, it is only summarised briefly here. The participants were presented via headphones with paired auditory stimuli generated by a computer with Waves MaxxAudio Pro sound controller software. Each pair of stimuli were separated by a silent gap of varied durations that changed during the experiment according to an adaptive-maximum-likelihood-based algorithm until the tested threshold had a probability of 95% inside a ± 5 ms interval around the currently estimated values^41^. The participant’s task was to verbally report the temporal order of two successive stimuli. TOJ included spatial and spectral tasks (Fig. 2). In the spatial TOJ task, stimuli were square-wave pulses (clicks) of 1 ms duration each, presented monaurally (i.e., one sound presented to one ear was followed by the presentation of the second sound to the other ear). In the spectral TOJ task, two 10 ms sinusoidal tones, differing in pitch (i.e., a low tone of 400 Hz and a high of 3000 Hz) were presented binaurally (i.e., each sound was presented to both ears at the same time). The loudness of the presented sounds was adjusted to participants individually and set at their comfortable listening level. The measurements for spatial and spectral tasks were conducted with each participant individually in two separate sessions in consecutive days. Each session lasted approximately 30 min and was preceded by a short introductory phase before each task. The outcome measure was auditory temporal-order threshold (TOT)—the shortest gap between two successive stimuli necessary for a participant to report correctly their temporal order (i.e., the before–after relation) with at least 75% correctness. The TOT values for the spatial and spectral tasks from these two sessions were averaged and analysed further for these two tasks separately.

#### Hundreds of milliseconds timing procedure

**Figure 3:**
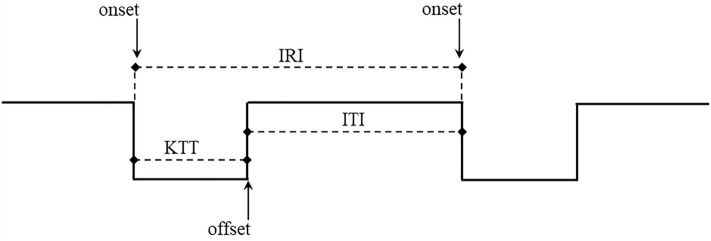
Schematic representation of three analysed tapping outcome measures: *IRI* inter-response interval, *KTT* key touching time, *ITI* inter-tap interval. Downward arrows mark initiation of the button press, whereas upward arrows indicate releasing the button.

Participants were seated comfortably with their forearms resting on a table. They were instructed to press with their right index finger, repetitively, as fast as possible, the indicated button placed on the response pad (Cedrus RB-740, Cedrus Corporation, San Pedro, CA). Responses were recorded using Presentation® software (Version 18.0, Neurobehavioral Systems, Inc., Berkeley, CA, USA). The task consisted of a short training phase followed by two consecutive regular finger tapping sessions of 30 s duration each (Session 1 and Session 2) and a 60 s rest between them. Tapping velocity and regularity during consecutive pressings were analysed using three components collected from each tap (displayed in Fig. 3):

- *Inter-response interval (IRI)* mean duration (in ms) of the period between onsets of two consecutive taps *(onset to onset*), reflecting the overall component of tapping velocity.
- *Key touching time (KTT)* mean duration (in ms) of contact between the index finger and the response button *(onset to offset)* in regular finger tapping sequences, reflecting the motor component in the execution of consecutive taps. In other words, the KTT reflects the time span between the end of agonistic and the beginning of antagonistic finger movements.
- *Inter-tap interval (ITI)* mean interval (in ms) between the offset of one tap and the onset of the next tap *(offset to onse*t), reflecting the time needed for planning and preparing consecutive movements.

In order to track changes of tapping rate and regularity across sessions, in further steps of analysis, the data obtained during consecutive tapping sequences in each session were divided into three parts (Part 1, 2, and 3), comprising 10 s intervals of consecutive tapping sequences.

## Results

### Tens of millisecond timing: spatial and spectral TOJ tasks

The participants’ performances in both TOJ tasks were characterised by high individual variability. For the spatial task, the measured TOT values were from 12 to 157 ms (mean TOT = 49, SD = 29), whereas in the spectral task from 15 to 390 (mean TOT = 97, SD = 75). The individual between-task differences in temporal order perception as well as underlying neural mechanisms were reported in detail in our previous studies^5,42^.

**Figure 4:**
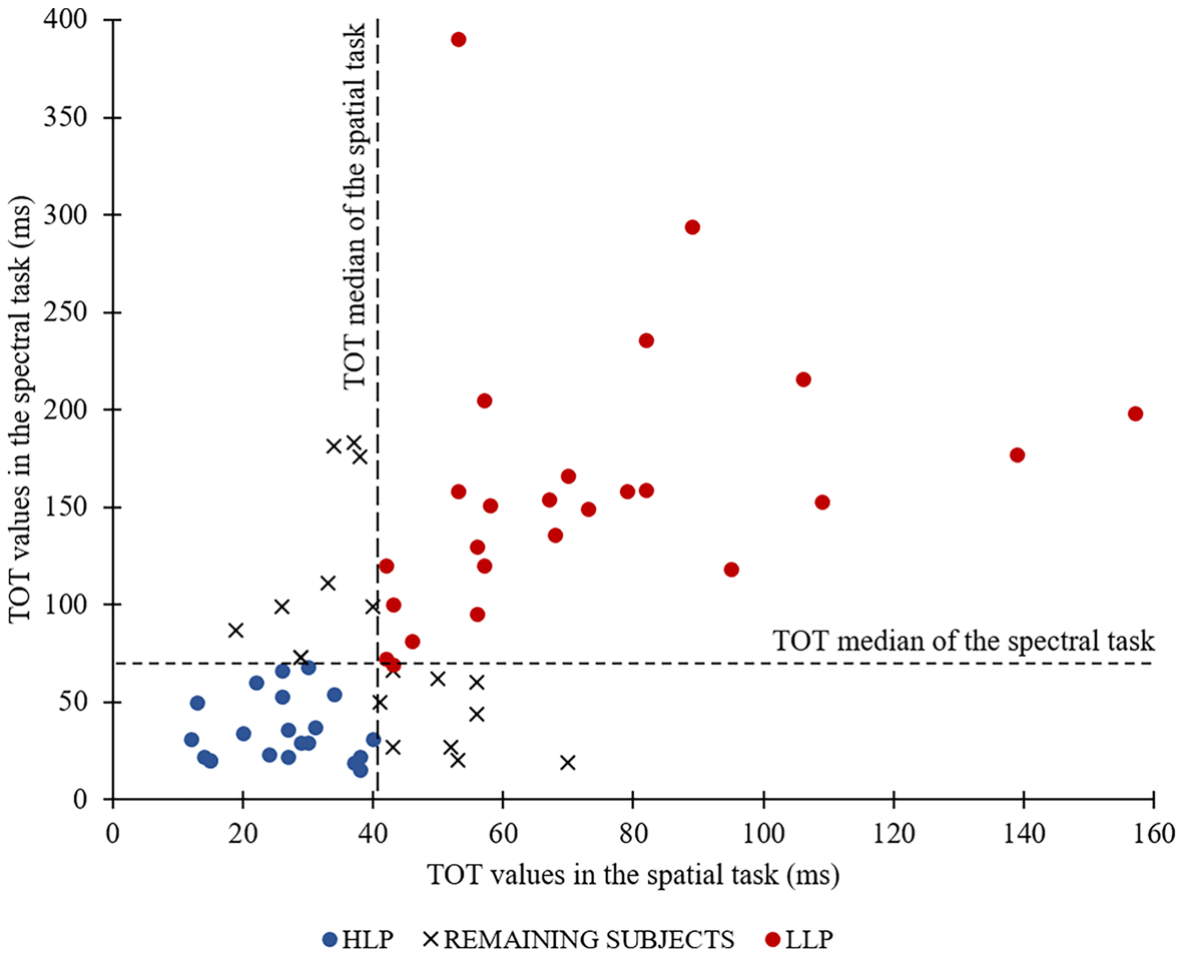
Scatter plot data presenting TOT values obtained in the spatial and spectral TOJ tasks in the initial participant sample. The vertical and horizontal dashed lines reflect the median TOT values in these two tasks, indicating four quartiles of obtained results. Blue dots reflect TOTs below the median values achieved in both tasks and red dots values above the medians; crosses reflect TOTs above the median in one task only—these participants were excluded from further analyses reported here.

Using Spearman rank correlation analysis, we found strong significant positive correlations between the TOT values obtained in these two tasks (r_s_ = 0.595; *p* < 0.001). Better performance in the spatial task (reflected in lower TOT) was accompanied by better performance in the spectral task. In other words, participants characterised by better temporal performance in one task also had better performance in the other task. Thus, the temporal performance measured in these two tasks may reflect the specific property of information processing in the tens of milliseconds time range reflecting, the general participant’s perceptual capacity, which seems to be independent of the given stimulus presentation mode (Fig. 4).

On the basis of these tasks, in the next step of data analysis, participants were classified into two groups according to the median values achieved in the initial sample (N = 64) in the spatial (Me = 42 ms) and spectral (Me = 73 ms) tasks. The group characterised by TOT values below these medians in both tasks (with better performance corresponding to lower TOT values) were classified as high-level performers (HLP); this group consisted of 21 participants. On the other hand, the group characterised by TOT above these medians in both tasks were classified as low-level performers (LLP); this group consisted of 25 participants. The remaining participants (N = 18) were characterised by mixed results (i.e., TOT values above/below the median in one task and below/above in the other) were removed from further analysis (Fig. 4). A similar classification procedure was also applied in our previous paper^2^ and participants included in the current study constitute a subgroup of those in the previous paper. Detailed characteristics of the groups are provided in Table 1.

**Table 1:** Characteristics of high-level performers (HLP) and low-level performers (LLP).

Group	n	Age (years)		Sex	TOT (mean in ms)	
		Range	Mean (SD)	(Female/male)	Spatial	Spectral
HLP	21	20–26	23 (2)	7/14	26	35
LLP	25	20–26	23 (2)	13/12	73	160

### Hundreds of millisecond timing: maximum tapping task

#### Outlier selection

Outlier identification was performed separately for each component by converting them to z-scores, using an exclusion criterion of z > 2.5 and z < − 2.5. Approx. 2% of outliers were excluded on this basis. Next, the mean values of the three tapping components (IRI, ITI, KTT) were calculated separately for Sessions 1 and 2, taking Parts 1, 2, and 3 into account.

#### Tapping velocity

Individual differences in tapping velocity in the selected groups were further tested with a 4-way analysis of variance (ANOVA 1) applied to the mean IRI. The tested variables were *Group* (HLP, LLP) and *Sex* (female, male) as between-subject variables, as well as *Session* (1, 2) and *Part* (1, 2, 3) as within-subject variables. Significant main effects were found for *Group* [F(1, 42) = 7.07; p = 0.011; η2 = 0.14] and *Part* [F(1.35, 56.49) = 104.72; p < 0.001; η2 = 0.71]. The variables *Session* and *Sex* and all interactions were nonsignificant. HLP showed shorter IRI (M_HLP_ = 158 ms) than did LLP (M_LLP_ = 187 ms), reflecting an 18% faster tapping rate in the former group. Furthermore, IRI increased progressively over the three tapping parts in both groups, reflecting 12% slowing of the tapping rhythm across consecutive parts, independently of the group (*p* < 0.001 for all between-part comparisons; i.e., between Part 1 vs 2, Part 2 vs 3, and Part 1 vs 3).

In parallel, the performance variability of participants’ responses was tested using 4-way analysis of variance (ANOVA 2) with the same factors as tested in ANOVA 1, but applied to the coefficients of variation obtained for particular IRI situations. In ANOVA 2, all factors tested, as well as interactions between these factors, were nonsignificant, indicating similar performance variability across groups, sex, sessions, and parts.

#### Relationship of the mental (ITI) and motor (KTT) components with tapping velocity

A 5-way ANOVA 3 was performed on the duration of the two main tapping components with *Group* (HLP, LLP) and *Sex* (female, male) as between-subject variables, as well as *Component* (ITI, KTT)*, Session* (1, 2) and *Part* (1, 2, 3) as the within-subject variables. ANOVA 3 revealed main effects of *Group* [F(1, 42) = 6.86; *p* = 0.012; η^2^ = 0.14]; *Part* [F(1.44, 60.41) = 102.53; *p* < 0.001; η^2^ = 0.71], whereas *Sex, Component*, and *Session* proved nonsignificant. Moreover, two interactions were significant: *Part* × *Component* [F(1.68, 70.41) = 5.55; *p* = 0.009; η^2^ = 0.12] and *Group* × *Component* × *Part* [F(1.68, 70.41) = 10.45; *p* < 0.001; *η*^2^ = 0.20].

**Figure 5:**
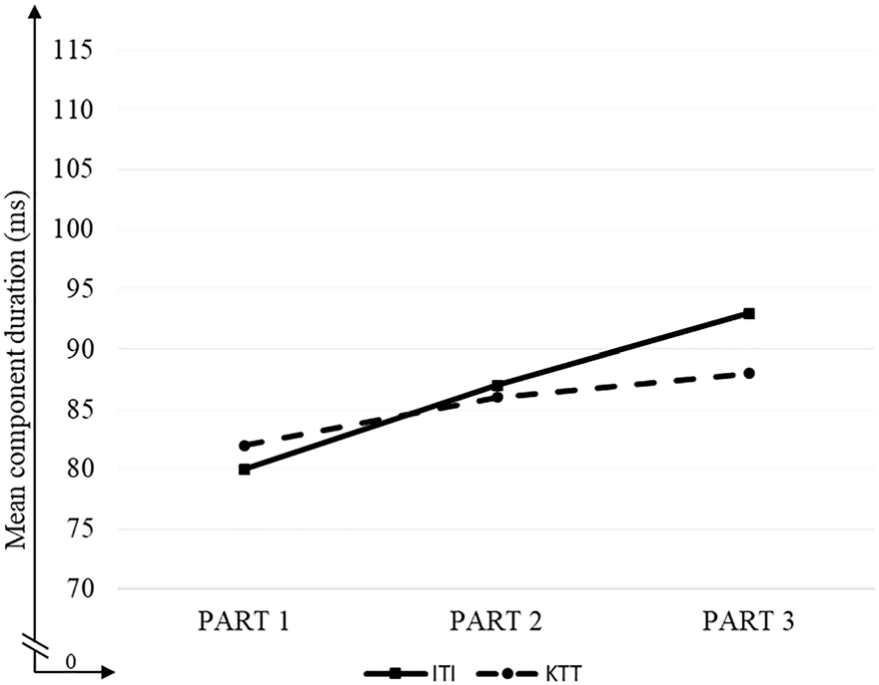
Averaged inter-tap interval (ITI) and key touching time (KTT) in consecutive Parts (1, 2, 3) of tapping sequences.

In general, HLP had shorter duration for both components than did LLP (*p* = 0.012). The component rate slowed down progressively in consecutive parts (*p* < 0.001 for Part 1 vs 2, Part 2 vs 3 and Part 1 vs 3). The *Component* factor proved nonsignificant. The 2-way *Component* × *Part* interaction resulted from different deceleration of components in consecutive parts, but it was more pronounced for ITI (16% deceleration) than for KTT (7%; Fig. 5). ITI increased consistently over consecutive parts (*p* < 0.001 for Part 1 vs 2, Part 2 vs 3 and Part 1 vs 3). On the other hand, KTT increased only between Part 1 versus 2 (*p* = 0.006) and Part 1 versus 3 (*p* < 0.001), being nonsignificant between Part 2 versus 3 (*p* = 0.078). The differences between ITI and KTT duration for particular parts were nonsignificant. These relations are illustrated in Fig. 5.

**Figure 6:**
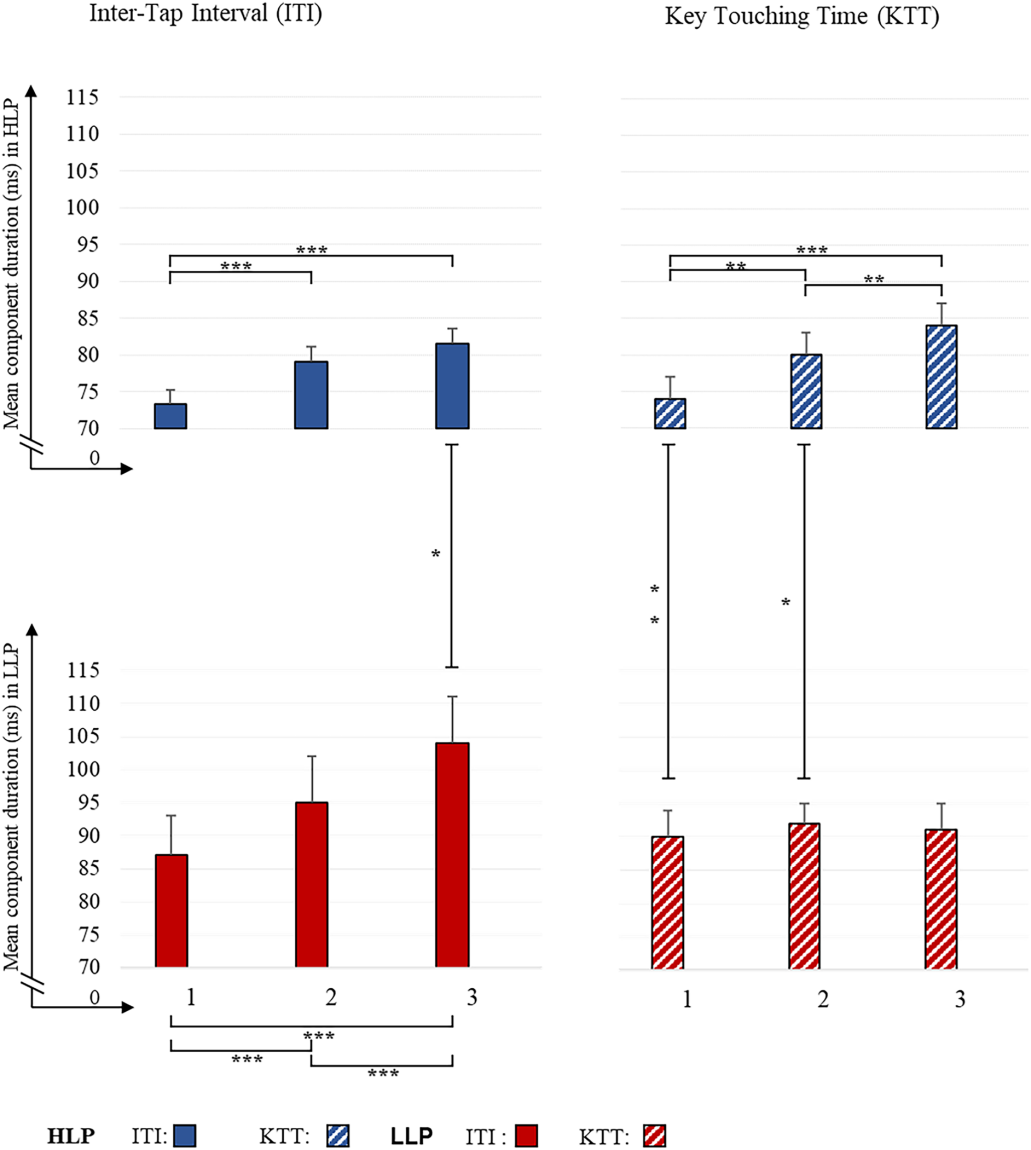
Average of inter-tap interval (ITI) and key touching time (KTT) components, plotted for both groups (HLP and LLP) and for the three Parts of task execution (1, 2, 3). Asterisks indicate significant differences: *p < 0.05; **p < 0.01; ***p < 0.001. Significant differences are marked with lines.

The above 2-way interaction was modified by *Group*, which was reflected in the 3-way *Group* × *Component* × *Part* interaction indicating a different pattern of deceleration for HLP and LLP over consecutive parts (Fig. 6). Such deceleration for HLP was significant for both components (11% and 14% between Part 1 and 3 for ITI and KTT, respectively), whereas in LLP it was significant only for ITI (20%), being nonsignificant for KTT (1%). Detailed data with significance values for particular comparisons are given in Fig. 6. Furthermore, in HLP, ITI was significantly shorter in only Part 3 (*p* = 0.012), whereas KTT was shorter in Part 1 (*p* = 0.004) and in Part 2 (*p* = 0.024) compared to LLP.

In parallel, the stability during repeated tapping performance was tested using 5-way analysis of variance (ANOVA 4) with the same factors as tested in ANOVA 3 but applied to the coefficients of variation obtained for ITI and KTT in particular situations. In ANOVA 4, all factors tested, as well as interactions, proved nonsignificant, indicating the similar precision of responses across groups, sex, components, sessions, and parts.

## Summary of results

The present study identified relationships between TIP in the tens of milliseconds time domain (TOJ tasks) and the hundreds of milliseconds time domain (maximum tapping task). In summary, HLP showed shorter IRI (M_HLP_ = 158 ms) than did LLP (M_LLP_ = 187 ms), reflecting an 18% faster tapping rate in the former group. Furthermore, IRI increased progressively in both groups over the three parts of the tapping task, reflecting a 12% slowing of the tapping rhythm across consecutive parts.

In HLP, a shorter duration of both tapping parameters (ITI and KTT) compared to LLP was observed. HLP and LLP had different patterns of deceleration in ITI and KTT over consecutive parts. Whereas both components significantly decelerated for HLP, LLP presented deceleration for ITI only, with deceleration being nonsignificant for KTT. Furthermore, a significant between-group difference for ITI was noticed after tempo stabilisation in HLP (only in Part 3), whereas it was observed for KTT in Part 1 and Part 2. In both groups, the differences in variability of performance were nonsignificant for all factors analysed.

## Discussion

The results of this study indicate a relationship between the efficiency of participants’ TIP performance across two different time domains with reference to perceptual and motor timing. These relations were evidenced for the perceptual TOJ task used to evaluate TIP performance in the tens of milliseconds domain and the motor tapping task used to assess TIP performance in the hundreds of milliseconds domain.

Based on the two TOJ task results (spatial and spectral; Fig. 2), which may be considered as a reliable indicator of TIP performance in the tens of milliseconds domain, groups of high-level performers (HLP) and low-level performers (LLP) were identified (Fig. 4). We may speculate that HLP are characterised by a higher pacemaker rate, which may result in higher accuracy of temporal order perception. In contrast, LLP may have a slower pacemaker rate, resulting in poorer performance of TOJ tasks. As hypothesised, HLP and LLP evidenced consistently divergent patterns of performance on the tapping task that indicates performance in the hundreds of milliseconds domain. Our study revealed that HLP had significantly shorter inter-response interval (IRI; M_HLP_ = 158 ms; i.e., a faster tapping rate) compared to LLP (M_LLP_ = 187 ms). Thus, individuals characterised by lower TOTs (better performance in TOJ tasks) manifested better performance on the hundreds of milliseconds level (i.e., faster tapping rate in the maximum tapping task). In contrast, participants who displayed higher TOTs (poorer performance on the TOJ tasks) had slower tapping rates. It is worth mentioning that the variability of performance indexed by the IRI in both groups studied here was similar.

Another important result was that, independently of the group, tapping rate (IRI) decelerated over the analysed parts—both HLP and LLP displayed slower tapping rates in consecutive parts. In our study, we disentangled the IRI component into inter-tap interval (ITI) and key touching time (KTT) to further explore the difference between HLP and LLP, taking into account the specificity of the processes underlying these components^31^. ITI is considered as a central timing component that plays a role in the mental planning of movement, which depends strongly on the speed of information processing controlled by the pacemaker rate. In contrast, KTT is considered as an automatized ballistic component and reflects the peripheral timing of the execution of particular motor acts^30,31^. It is important to note that both mental and motor components (ITI and KTT) were shorter in HLP, which reflects their higher speed of information processing and better motor execution compared to LLP. Again, the differences in variability of these processes were similar in both groups. However, divergent patterns for both ITI and KTT were observed in consecutive parts in two groups.

In HLP, the mental component began to stabilise in Part 2 (nonsignificant difference between Parts 2 and 3, Fig. 6), while in LLP it decelerated over consecutive parts. Significant between-group differences emerged in Part 3 due to an increase of ITI in LLP (Fig. 6). It has been assumed that, in addition to HLP having significantly shorter ITIs, they synchronised faster with their pacemaker rate. This could be due to the specificity of the general functioning of the hypothesised internal clock, which is consistent with the faster rate of the pacemaker and more efficient regulation of its speed^43^. In LLP, longer ITI may be associated with the lower speed of their pacemaker. Progressive ITI deceleration may indicate difficulties in synchronisation with the internal clock, suggesting a less stable clock ticking frequency^28^.

Furthermore, the shorter KTT in HLP progressively decelerated across consecutive parts. The most common explanation in the literature of the increase of KTT is that it is due to muscle fatigue reflected in reduced temporal control of motor acts^44^. A similar observation was reported by Hubel^24^, who noticed a great increase of KTT over a 30 s tapping period in young individuals. As in our HLP group, these young individuals started from a faster tapping rate (KTT) compared to elderly participants, which caused them to be more prone to hand muscle fatigue^24^. In LLP, KTT was relatively stable across consecutive parts, indicating that their tapping rates were closer to their optimum speed^24^.

To sum up, we found that participants classified as HLP and LLP based on the TOJ tasks were characterised by better or poorer tapping task performance, respectively. However, the significant HLP versus LLP differences resulted from the motor component (shorter in HLP) in the early stages and mental component (shorter in HLP) in the last stage of the tapping task.

Our findings suggest that classification of participants based on their TIP performance in the tens of milliseconds domain with our TOJ tasks could be a good predictor of their performance in higher time domain.

The cross-domain relations identified here with the TOJ and tapping tasks provide solid arguments for the existence of a common timing mechanism, which may operate independently of time domains and timing systems (perceptual vs motor). This observation is in line with Pöppel’s hierarchical model of TIP^7^, in which the tens of milliseconds domain provides the basic framework for temporal processing on the higher levels.

To understand the potential mechanisms underlying the perception of event succession, we can refer to the basic processes in the nervous system measured by oscillatory brain activity. Electrophysiological studies have indicated that perception of succession is controlled by an internal timing mechanism operating in a time window of some tens of milliseconds, probably implemented in neuronal gamma band oscillations with a periodicity of ca. 40 Hz^45,46^; the before–after relation can be properly identified only if two stimuli occur in at least two successive oscillatory periods^7^. Thus, gamma band oscillations correspond to the TOT values in the TOJ tasks applied in our study. Hence, there is strong evidence that spontaneous, or stimulus triggered, gamma band oscillations correspond in duration to the TOT.

The beta rhythm is another candidate for playing a fundamental role in our functioning in the tens and hundreds of milliseconds domains. It has been shown that, during the TOJ task, beta oscillations occur noticeably and, furthermore, are significantly stronger before TOJ trials performed correctly^35^. Thus, beta rhythm oscillations of a frequency of approx. 14–30 Hz (i.e., one period of ca. 30–70 ms duration) contribute to the timing mechanism in the tens of milliseconds domain. Furthermore, one MEG study found that when participants passively listen to auditory beats, there occurs coherence in the beta band between auditory and motor regions such as the SMA, cerebellum, and pre- and post-central gyri^47^. Furthermore, Bartolo and Merchant^36^, registered local field potentials from the putamen during a synchronisation–continuation tapping task. They found that beta oscillations were observed in synchronisation and continuation phases of tasks that operate in the hundreds of milliseconds domains. In summary, we may argue that high-frequency oscillations (gamma and beta) are associated with TIP and their coupling is linked with auditory–motor neural communication^48^.

Further evidence for the presence of a common timing mechanism comes from fMRI studies. Merchant^42^ proposed a mechanism represented in the main core timing network (cortico–thalamic–basal ganglia circuits and the supplementary motor area) and context-specific brain structures. Thus, TIP depends on the cooperation of multiple brain areas. Meta-analyses conducted by Nani et al.^37^ confirmed that, in addition to distinct timing mechanisms that may operate on different timescales, shared core neural structures control cross-domain temporal processing. Taken together, perceptual and motor tasks addressing different time domains may be controlled by cerebral areas common to TIP (i.e., the insula, superior, frontal, and medial frontal gyri, precentral gyrus, cingulate gyrus, superior gyrus, claustrum, putamen, and caudate body;^37^).

The study has some limitations. Sorting the participants into two groups (HLP, LLP) resulted in small sample sizes; nonetheless, the sizes were sufficient for the analyses performed (a-priori power analysis). The second stage, in which two separate components of the tapping task were analysed, had more exploratory approach. However, in future studies, we plan to verify our results on larger samples of participants to allow us to apply more sophisticated statistical analyses as well as to analyse cross-domain relations for participants characterised by average temporal performance.

Despite considering formal musical education as an exclusion criterion, there are some other factors (e.g., musical, dancing, or sporting activities) that may influence the performance of timing tasks and were not controlled in our study. We recommend that future studies take into account these variables, as they may potentially affect the participants’ temporal ability.

To conclude, the results of the present study suggest that a common timing mechanism in the tens of milliseconds domain may also regulate TIP in the hundreds of milliseconds domain, regardless of the task specificity (perceptual vs motor). Thus, a person's performance in the tens of milliseconds domain may indicate their performance in the hundreds of milliseconds domain. Hence, reliable measurement of the tens of milliseconds domain might help us to draw reasonable conclusions about TIP performance on higher levels.

## Data Availability

The datasets generated and/or analysed during the current study are available from the corresponding author on request.

## Abbreviations

TIP: Temporal information processing
TOJ: Temporal-order judgement
TOT: Temporal-order threshold
IRI: Inter-response interval
KTT: Key touching time
ITI: Inter-tap interval
HLP: High-level performers
LLP: Low-level performers
